# [Corrigendum] miR‑181a‑5p inhibits the proliferation and invasion of drug‑resistant glioblastoma cells by targeting F‑box protein 11 expression

**DOI:** 10.3892/ol.2025.14965

**Published:** 2025-03-05

**Authors:** Xueyan Wen, Songrong Li, Mengchan Guo, Hongzhan Liao, Yongmin Chen, Xi Kuang, Xiaoping Liao, Lin Ma, Qifu Li

Oncol Lett 20: 235, 2020; DOI: 10.3892/ol.2020.12098

Following the publication of the above article, an interested reader drew to the authors’ attention that the western blot data (both the control GAPDH and the FBXO11 protein bands) in Fig. 3C on p. 6 were strikingly similar to data which had previously appeared in an article published in the journal *Oncotarget*, albeit one of the authors (Hongzhan Liao) was featured as an author on both papers. After having re-examined their original data, the authors realized that the wrong data had inadvertently been included in this figure part.

The revised version of Fig. 4, now featuring the correct data for the GAPDH and the FBXO11 experiments in Fig. 3C, is shown on the next page. Note that the errors made in assembling this figure did not affect the overall conclusions reported in the paper. All the authors agree with the publication of this corrigendum, and are grateful to the Editor of *Oncology Letters* for allowing them the opportunity to publish this. The authors also apologize to the readership for any inconvenience caused.

## Figures and Tables

**Figure 3.**
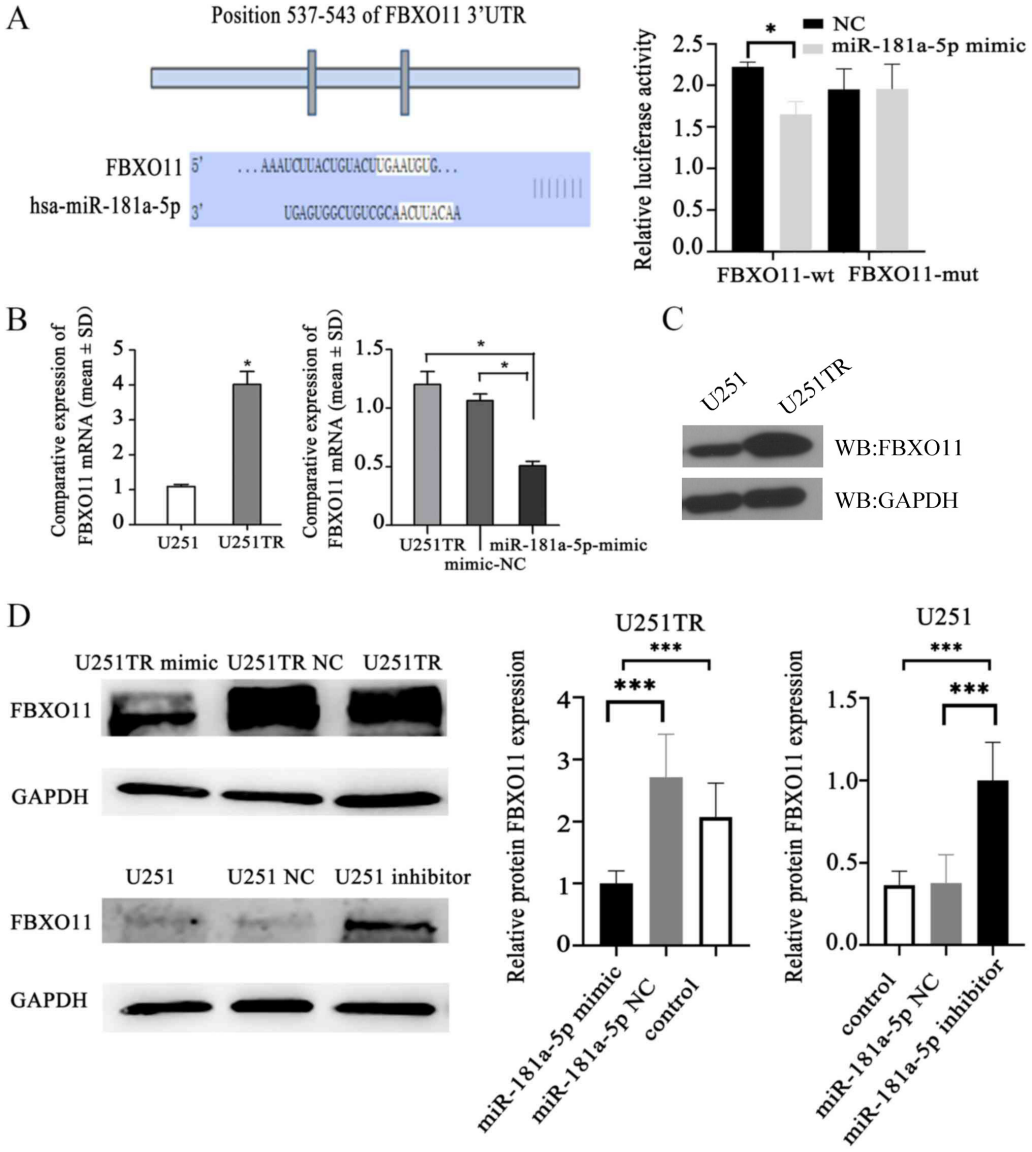
miR-181a-5p targeting FBXO11. (A) Map of FBXO11 and miR-181a-5p target sites. In the 3’-UTR FBXO11 non-coding region. The luciferase activity of U251 cells co-transfected with miR-181a-5p mimic and wt FBXO11 3’-UTR was lower compared with that of NC cells in FBXO11-wt group. *P<0.05. (B) The left panel shows the expression of FBXO11 mRNA in glioma and drug-resistant cell lines. The right panel shows that upregulation miR-181a-5p inhibits the expression levels of FBXO11 mRNA in glioma-resistant cells. *P<0.05. (C and D) Western blot assays were performed to determine (C) FBXO11 expression in normal glioma cells and drug-resistant glioma cells, and (D) miR-181A-5P targeting FBXO11. FBXO11 expression was downregulated in drug-resistant glioma cells transfected with miR-181a-5p mimic. FBXO11 expression was upregulated in glioma cells transfected with miR-181a-5p inhibitor. ***P<0.001. miR, microRNA; UTR, untranslated region; FBXO11, F-Box protein 11; NC, negative control; wt, wild-type.

